# Acetaminophen use and prognosis in cancer patients treated with immune checkpoint inhibitors: evidence from a meta-analysis

**DOI:** 10.3389/fimmu.2025.1682686

**Published:** 2025-11-21

**Authors:** Yuxuan Lin, Yonghe Liao, Jinhai Shen

**Affiliations:** 1 Department of Pharmacy, Guangxi Hospital Division of The First Affiliated Hospital, Sun Yat-sen University, Nanning, China; 2 School of Pharmaceutical Science, Guangxi Medical University, Nanning, China; 3 Department of Pharmacy, The Second Affiliated Hospital of Guangxi Medical University, Nanning, China; 4 State Key Laboratory of Natural Medicines, China Pharmaceutical University, Nanjing, Jiangsu, China; 5 Center for New Drug Safety Evaluation and Research, China Pharmaceutical University, Nanjing, Jiangsu, China; 6 School of Basic Medicine and Clinical Pharmacy, China Pharmaceutical University, Nanjing, Jiangsu, China

**Keywords:** acetaminophen, cancer, immune checkpoint inhibitors, prognosis, meta-analysis

## Abstract

**Background:**

Emerging studies have investigated the association between acetaminophen (APAP) use and clinical outcomes in cancer patients receiving immune checkpoint inhibitors (ICIs), but their findings remain inconsistent. This meta-analysis aims to systematically synthesize available evidence to clarify this relationship and provide evidence-based guidance for clinical practice.

**Methods:**

A systematic literature search was performed to identify studies comparing prognostic outcomes between APAP users and non-users among cancer patients treated with ICIs. Eligible studies were required to report hazard ratios (HRs) for overall survival (OS) and/or progression-free survival (PFS) with 95% confidence intervals (CIs). Meta-analyses were conducted to derive pooled effect estimates. Funnel plots and Egger’s test were used to assess publication bias, and sensitivity analyses via a leave-one-out approach were performed to evaluate the robustness of results.

**Results:**

Five studies encompassing 7 cohorts and 2,349 patients (1,306 APAP users and 1,043 non-users) were included. Pooled analyses revealed that concomitant APAP use was significantly associated with shorter OS (HR: 1.29; 95% CI: 1.16–1.44) and PFS (HR: 1.27; 95% CI: 1.12–1.43), as well as a trend toward a lower objective response rate (RR: 0.78; 95% CI: 0.60–1.00). No significant publication bias was detected, and sensitivity analyses confirmed the robustness of these findings.

**Conclusion:**

Current evidence indicates that APAP use is associated with poorer prognosis in cancer patients treated with ICIs. These results may inform clinical guidelines regarding concomitant APAP and ICI use. Further randomized controlled trials are warranted to validate these observations and establish causal relationships.

**Systematic Review Registration:**

https://www.crd.york.ac.uk/prospero/, identifier CRD420251118489.

## Introduction

Immune checkpoint inhibitors (ICIs) have revolutionized cancer treatment by reactivating cytotoxic T-cell responses against malignant cells (1). By targeting negative regulatory pathways such as programmed cell death protein 1 (PD-1), programmed death-ligand 1 (PD-L1), and cytotoxic T-lymphocyte–associated antigen 4 (CTLA-4), ICIs have demonstrated durable clinical benefits and survival advantages in a variety of tumor types (2). Despite these breakthroughs, a considerable proportion of patients fail to achieve sustained responses, underscoring the need to identify factors that influence immunotherapy efficacy.

One increasingly recognized yet underexplored factor is the role of concomitant medications in modulating ICI outcomes (3–5). Several classes of non-oncologic agents—including corticosteroids, antibiotics, and proton pump inhibitors—have been implicated in dampening ICI efficacy, either through direct immunosuppression or via disruption of host-microbiota interactions (6–9). Acetaminophen (APAP), one of the most frequently used antipyretic and analgesic agents in oncology, is widely regarded as safe and well-tolerated (10). However, emerging evidence suggests that APAP may exert unintended immunomodulatory effects, raising concern about its potential to compromise antitumor immune responses (11).

Mechanistic studies have shown that APAP can inhibit T-cell proliferation, suppress interferon-gamma (IFN-γ) production, and promote regulatory T cell (Treg) expansion—features that collectively impair cytotoxic immunity (11). In addition, APAP has been reported to elevate levels of immunosuppressive cytokines such as interleukin-10 (IL-10) and to attenuate antigen-presenting cell function (11). Clinical observations echo these findings: APAP exposure has been associated with reduced antibody responses to viral infections and vaccinations, prompting international health agencies to advise caution regarding its routine use in immunologically sensitive contexts (12).

Despite these signals, clinical data on APAP use during ICI therapy remain limited and inconsistent. Retrospective studies examining the association between APAP exposure and ICI outcomes have yielded conflicting results (11, 13). Given the ubiquity of APAP use in cancer care—and the increasing reliance on ICIs as a cornerstone of systemic therapy—clarifying this relationship is of substantial clinical importance.

To address this gap, we conducted a systematic review and meta-analysis of studies evaluating the prognostic impact of APAP use in cancer patients treated with ICIs. By integrating data across cohorts and tumor types, we aimed to quantify the association between APAP exposure and survival outcomes, assess potential biases, and provide evidence-based guidance for the supportive care of patients undergoing immunotherapy.

## Methods

### Study registration

This meta-analysis was conducted in accordance with the Preferred Reporting Items for Systematic Reviews and Meta-Analyses (PRISMA) statement (14). The protocol has been registered in the International Prospective Register of Systematic Reviews (PROSPERO) (identifier: CRD420251118489).

### Data sources and search strategy

A systematic literature search was conducted using PubMed, Web of Science, and Embase, covering all records from their inception to August 2, 2025. The search strategy combined terms related to acetaminophen (e.g., acetaminophen, paracetamol, N-acetyl-para-aminophenol) and immune checkpoint inhibitors (e.g., immune checkpoint inhibitors, PD-1 inhibitors, PD-L1 inhibitors, CTLA-4 inhibitors, pembrolizumab, nivolumab, atezolizumab, ipilimumab), as well as terms pertaining to prognostic outcomes. The complete search strategies for each database are detailed in **Supplementary Table 1**.

### Eligibility criteria and study selection

Studies were deemed eligible if they met the following criteria: (i) assessed the prognostic impact of APAP use in patients receiving ICIs, with comparator groups comprising patients not receiving APAP (concomitant use of other medications permitted); and (ii) reported hazard ratios (HRs) with corresponding 95% confidence intervals (CIs) for overall survival (OS) and/or progression-free survival (PFS). Eligible study designs included randomized controlled trials (RCTs) and observational cohort studies. Exclusion criteria encompassed review articles, case reports, preclinical studies, studies not evaluating APAP in relation to clinical outcomes, and those comparing high-dose *versus* low-dose APAP without a non-APAP control group.

The literature was managed and deduplicated using EndNote. Two independent reviewers conducted the initial screening based on titles and abstracts, followed by full-text evaluations for final inclusion. Any discrepancies were resolved through discussion with a third reviewer.

### Data extraction and quality assessment

Two reviewers independently extracted data using a standardized data collection form. Any discrepancies were resolved through consensus or, if necessary, by consultation with a third reviewer. The following information was extracted:

Study characteristics: First author, publication year, country, study design, and sample size.
Patient characteristics: Cancer type, ICI regimen, and number of patients receiving APAP.
Outcome data: HRs with corresponding 95% CIs for OS and PFS, as well as objective response rate (ORR). When both unadjusted and adjusted HRs were reported, adjusted estimates controlling for potential confounders were prioritized.

The methodological quality of the included studies was assessed using the Newcastle–Ottawa Scale (NOS) for cohort studies, which evaluates study quality across three domains: selection of study groups, comparability of groups, and ascertainment of outcomes (maximum score = 9 stars). Studies with a NOS score ≥ 7 were be considered high quality (15).

### Statistical analysis

Meta-analyses were conducted using R software (version 4.5.1). Heterogeneity among studies was assessed using the *I²* statistic and Cochran’s *Q* test. An *I²* value greater than 50% along with a *Q*-test *p*-value less than 0.10 was considered indicative of significant heterogeneity. The DerSimonian–Laird random-effects model was applied when substantial heterogeneity was present, whereas the Mantel–Haenszel fixed-effects model was used for homogeneous data. Pooled effect sizes were calculated for each outcome. HRs with 95% CIs were used for OS and PFS. Odds ratios (OR) with 95% CIs were used for ORR. Publication bias was assessed using funnel plots and Egger’s regression test, with a *p*-value less than 0.05 indicating significant bias. Sensitivity analyses were performed by sequentially excluding each study to evaluate the robustness of the results. All statistical tests were two-sided, and a *p*-value less than 0.05 was considered statistically significant.

## Results

### Study selection

**Figure 1:**
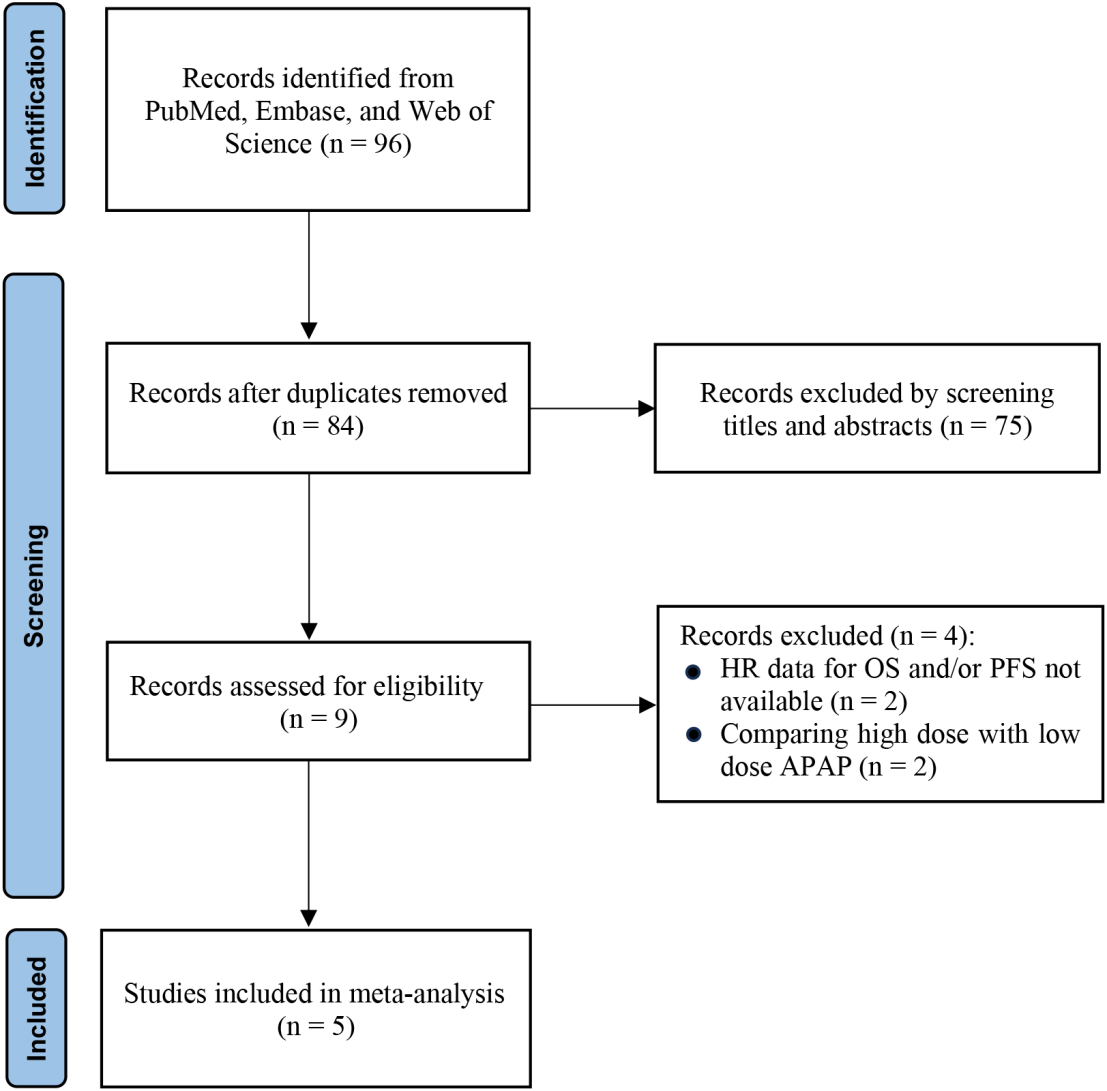
PRISMA flow diagram of study selection. Flowchart summarizing the literature search and selection process according to PRISMA 2020 guidelines. The diagram shows the number of records identified, screened, excluded (with reasons), and finally included in the meta-analysis.

The systematic literature search initially identified 96 potentially relevant records. After removing duplicates, 84 unique publications remained for preliminary eligibility assessment. Title and abstract screening led to the exclusion of 75 articles that were not relevant to the research objectives. As a result, 9 articles proceeded to full-text evaluation. Following rigorous application of the predefined inclusion criteria, 5 studies were deemed eligible and included in the final quantitative synthesis (11, 13, 16–18). The complete study selection process is illustrated in **Figure 1**.

### Characteristics of the included studies

All five included studies employed retrospective cohort designs, collectively comprising seven independent cohorts and a total of 2,349 patients (1,306 APAP users and 1,043 non-users). These studies were published between 2022 and 2025 and represented diverse geographic regions, including France (4 cohorts), China (Hong Kong; 1 cohort), Italy (1 cohort), Japan (1 cohort), and one multinational cohort. Considerable heterogeneity was observed in cohort sizes, which ranged from 34 to 753 patients, and in the prevalence of APAP exposure, which varied from 26.0% to 74.8%.

The included cohorts investigated a range of cancer types. Four focused exclusively on non-small cell lung cancer (NSCLC), one on renal cell carcinoma (RCC), and two included patients with various solid tumors. ICI regimens consisted of anti-PD-1/PD-L1 monotherapy (e.g., nivolumab, pembrolizumab, atezolizumab) or combination therapy with anti-CTLA-4 agents (e.g., ipilimumab). Detailed study characteristics are summarized in **Table 1**.

**Table 1:** Characteristics of the included studies.

Study	Study design	Country	APAP user /Sample size (%)	Cancer type	ICIs used	HR for OS (95% CI)	HR for PFS (95% CI)	ORR (presence *vs.* absence)	NOS
Bessede et al., 2022 CheckMate 025 cohort (11)	Retrospective cohort study	International	101/392 (25.8)	RCC	Nivolumab	1.49 (1.14-1.92)	NA	NA	9
Bessede et al., 2022 BIP cohort (11)	Retrospective cohort study	France	17/34 (50.0)	Multiple solid tumors	Anti-PD-L1 (monotherapy or combined with anti-CTLA-4)	1.43 (0.61-3.33)	1.59 (0.76-3.33)	0 *vs.* 29.4%	
Bessede et al., 2022 PREMIS cohort (11)	Retrospective cohort study	France	167/297 (56.2)	Multiple solid tumors	Anti-PD-1, anti-PD-L1, or combination of immunotherapies	1.78 (1.18-2.86)	1.43 (1.07-1.91)	20.7% *vs.* 28.9%	
Ye et al 2023 (16)	Retrospective cohort study	China (Hong Kong)	428/572 (74.8)	LC	NA	1.32 (1.04-1.64)	NA	NA	9
Nelli et al., 2024 (17)	Retrospective cohort study	Italy	53/176 (29.5)	NSCLC	Pembrolizumab	1.20 (0.83-1.73)	1.29 (0.91-1.83)	NA	9
Yamada et al 2024 (13)	Retrospective cohort study	Japan	33/127 (26.0)	NSCLC	Pembrolizumab, atezolizumab, nivolumab, and ipilimumab	0.91 (0.46-1.79)	1.23 (0.78-1.95)	60.6% vs. 61.7%	8
Gobbini et al., 2025 (18)	Retrospective cohort study	France	507/753 (67.3)	NSCLC	Nivolumab	1.19 (1.01-1.40)	1.21 (1.03-1.41)	NA	9

APAP, Acetaminophen; ICIs, immune checkpoint inhibitors; HR, hazard ratio; OS, overall survival; PFS, progression-free survival; ORR, objective response rate; LC, lung cancer; NSCLC, non-small cell lung cancer; RCC, renal cell carcinoma; NA, not available; PD-1, programmed cell death-1; PD-L1, programmed cell death 1 ligand; CTLA-4, cytotoxic T lymphocyte-associated antigen 4; NOS, Newcastle-Ottawa Scale.

Methodological quality, as assessed using the NOS, was uniformly high. Six cohorts received the maximum score of 9 out of 9, and one cohort received a score of 8, as presented in **Supplementary Table 2**.

### APAP use and prognosis in patients receiving ICIs

**Figure 2:**
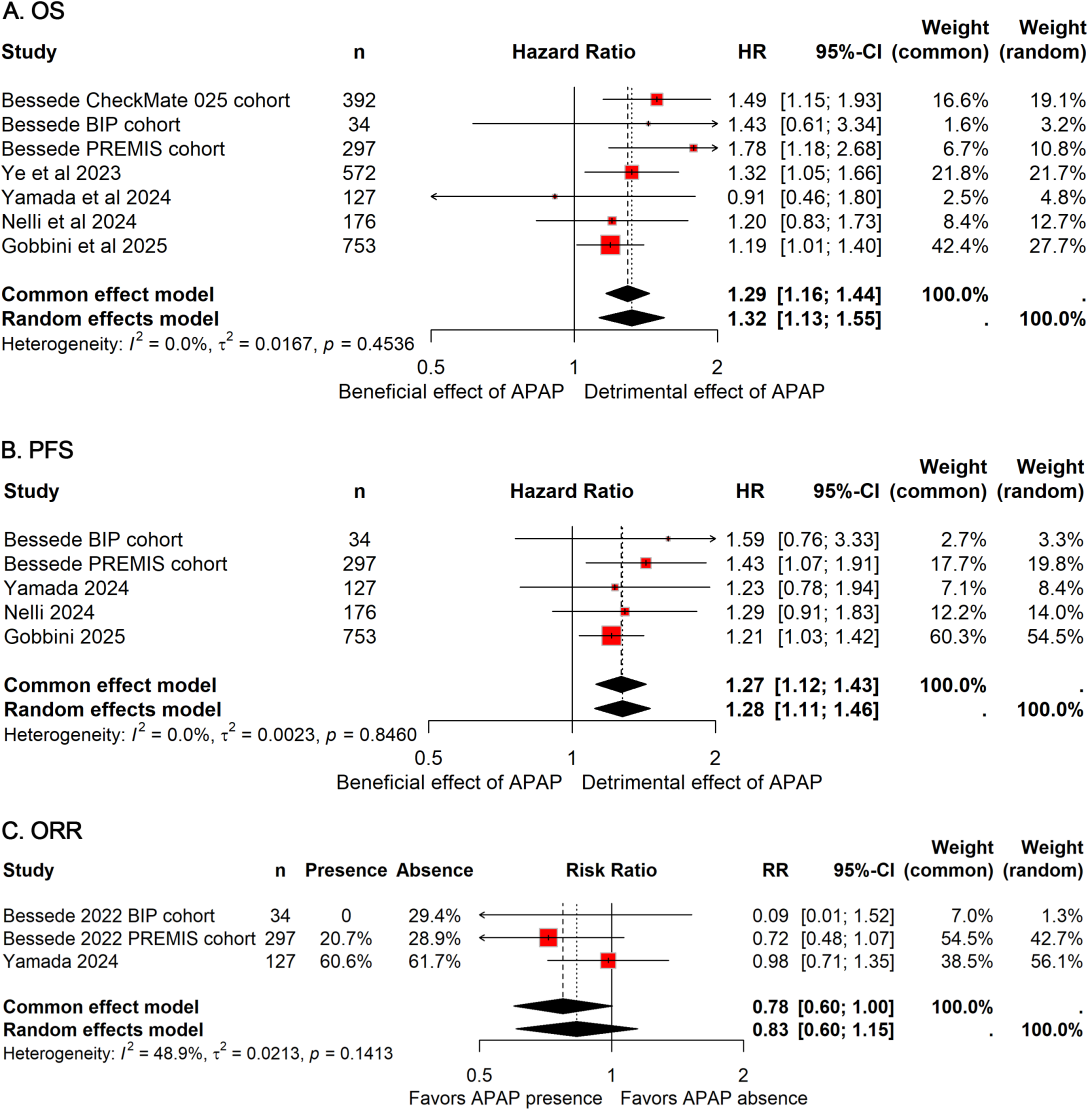
Forest plots of the prognostic impact of APAP use in patients receiving ICIs. Pooled HRs for OS **(A)** and PFS **(B)**, and pooled OR for ORR **(C)** are shown. Squares represent individual study estimates, with size proportional to study weight; horizontal lines indicate 95% CIs. Diamonds represent pooled estimates derived from random-effects models. HRs greater than 1 or OR less than 1 indicate worse outcomes in APAP users compared with non-users.

All studies included in this meta-analysis comprehensively examined the association between APAP use and OS in patients treated with ICIs, encompassing a total of 2,349 patients (APAP users: 1,306; non-users: 1,043). Notably, no heterogeneity was observed across studies (*I²*=0%), allowing for the application of a fixed-effect model to estimate the pooled HR. The analysis demonstrated that APAP use was significantly associated with a 29% increased risk of death (HR: 1.29; 95%CI: 1.16–1.44; **Figure 2A**), suggesting a potential detrimental effect of APAP on the efficacy of ICIs.

Five cohorts, including 1,387 patients (APAP users: 777; non-users: 610), assessed the impact of APAP use on PFS (11, 13, 17, 18). Consistent with OS findings, no heterogeneity was detected (*I²*=0%), and a fixed-effect model was utilized. The pooled results indicated a 27% reduction in PFS among patients receiving APAP concomitantly with ICIs (HR: 1.27; 95% CI: 1.12–1.43; **Figure 2B**), further supporting the notion that APAP use may negatively influence survival outcomes in this population.

Regarding ORR, data from three cohorts comprising 458 patients (APAP users: 217; non-users: 241) were analyzed (11, 13). The combined OR suggested a non-significant trend toward reduced ORR with concurrent APAP use compared to non-use (OR: 0.78; 95% CI: 0.60-1.00; **Figure 4C**).

### Publication bias and sensitivity analysis

**Figure 3:**
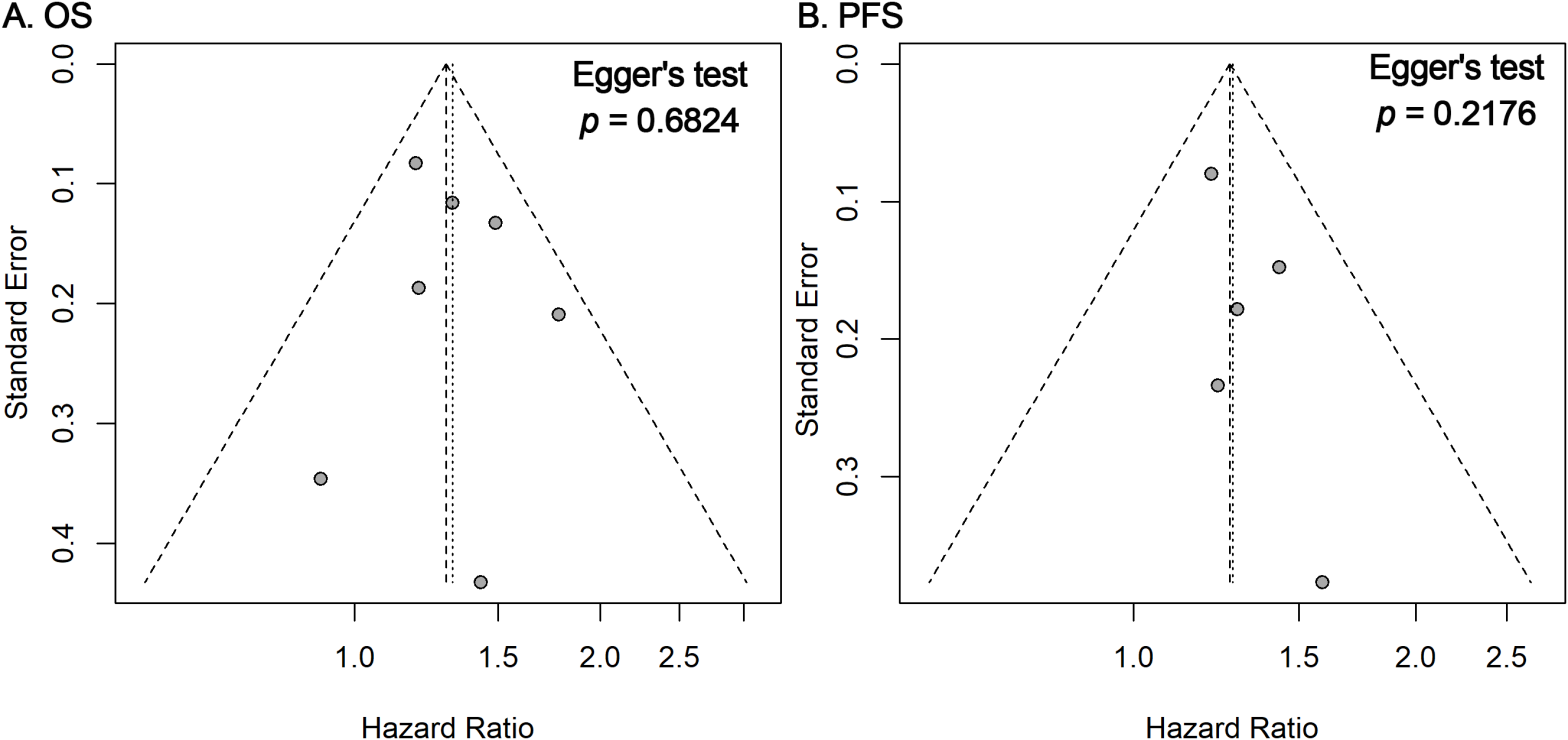
Funnel plots and Egger’s tests for publication bias. Funnel plots for OS **(A)** and PFS **(B)** outcomes showing the distribution of study-specific log HRs against their standard errors. Visual symmetry suggests a low likelihood of publication bias, which was further evaluated using Egger’s regression test (*p*-values shown).

**Figure 4:**
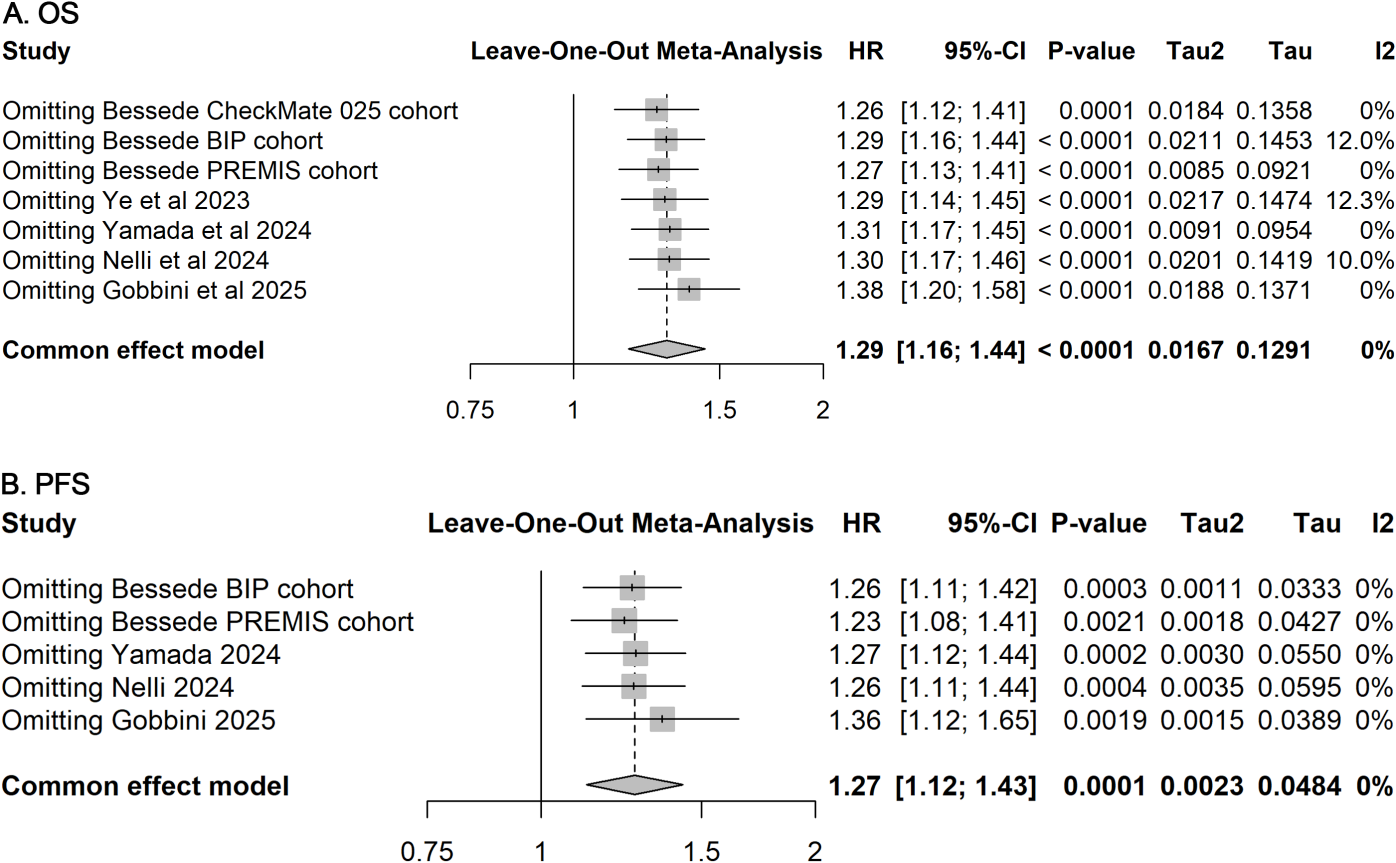
Sensitivity analyses for included studies on OS and PFS. Leave-one-out sensitivity analyses assessing the influence of each individual study on the pooled HR for OS **(A)** and PFS **(B)**. The stability of pooled estimates upon sequential exclusion of single studies indicates the robustness of the overall results.

Funnel plots and Egger’s regression tests revealed no significant publication bias for OS and PFS outcomes (**Figure 3**). Furthermore, sensitivity analyses employing a leave-one-out method confirmed the stability and robustness of the pooled estimates for both OS and PFS (**Figure 4**).

## Discussion

This meta-analysis consolidates evidence from 2,349 patients across seven cohorts, revealing a consistent association between APAP use and diminished clinical outcomes in cancer patients receiving ICIs. The observed HRs—1.29 for OS and 1.27 for PFS—translate to a nearly 30% increased risk of death or disease progression among APAP-exposed individuals. These findings assume critical significance in the context of immuno-oncology, where ICIs rely on robust T-cell-mediated antitumor responses, and even subtle immune modulation by concomitant medications can undermine therapeutic efficacy. The homogeneity of effects across diverse populations (*I²*=0%) and robustness to sensitivity analyses further underscore the clinical relevance of this association.

Although no significant statistical heterogeneity was detected, underlying variability in patient characteristics, tumor type, and APAP exposure definitions may have contributed to subtle clinical differences among studies. For example, four included cohorts exclusively enrolled NSCLC patients, while others involved RCC or mixed tumor populations, potentially influencing immune responsiveness. Moreover, some studies quantified plasma APAP levels, reflecting direct systemic exposure, whereas others relied on prescription data, which may underestimate biologically relevant dosing. Despite these variations, the direction of effect remained remarkably consistent across cohorts, suggesting a genuine association rather than a population-specific artifact. Future prospective analyses with standardized exposure definitions are warranted to validate this relationship.

The biological plausibility of the detrimental impact of APAP on ICI efficacy is supported by converging mechanistic evidence that elucidates how APAP interferes with the immune pathways targeted by checkpoint blockade. The therapeutic activity of ICIs depends on restoring cytotoxic CD8^+^ T-cell function and overcoming immunosuppressive barriers within the tumor microenvironment. APAP appears to counteract these processes through several complementary mechanisms. First, APAP fosters an immunosuppressive milieu by promoting regulatory T-cell (Treg) expansion and elevating interleukin-10 (IL-10) levels (11). These expanded Tregs further suppress effector T-cell proliferation and cytotoxic activity, partly through the release of inhibitory cytokines such as TGF-β (19). This immune shift toward tolerance has been observed in patients with detectable plasma APAP at ICI initiation, who exhibited increased circulating Tregs and IL-10 concentrations correlated with inferior survival outcomes (11). Second, APAP directly impairs effector T-cell function by suppressing interferon-gamma (IFN-γ) production—a key cytokine essential for antitumor immunity, antigen presentation, and tumor cell killing (11, 20). Experimental studies have demonstrated that APAP reduces IFN-γ secretion in anti–PD-1–stimulated peripheral blood mononuclear cells, indicating a direct antagonistic effect on the immune activation intended by PD-1 blockade (11). Beyond this, emerging evidence suggests that APAP may disrupt mitochondrial function and cellular energy metabolism, potentially compromising the bioenergetic demands of T-cell activation and proliferation, though this pathway warrants further investigation in the context of immunotherapy (21–23). Furthermore, APAP has been shown to induce neutrophil extracellular trap (NET) formation (24). These NETs not only hinder T-cell infiltration and cytotoxic function but may also promote tumor progression (25). In APAP-induced liver injury models, NETosis has been associated with AIM2 inflammasome activation and inflammatory cell death (PANoptosis), potentially exacerbating tissue injury and reinforcing an immunosuppressive microenvironment (26). This mechanism, already linked to reduced response to neoadjuvant chemoimmunotherapy in NSCLC (24), represents a novel physical and functional barrier through which APAP may diminish immunotherapy efficacy. Collectively, these findings highlight that APAP compromises multiple arms of antitumor immunity—including amplification of immunosuppressive circuits, suppression of effector T-cell signaling, potential disruption of T-cell metabolism, and induction of NET-mediated barriers—offering a coherent and multifaceted biological rationale for the poorer survival outcomes observed in APAP-exposed patients.

A critical consideration in interpreting these findings is the potential for confounding by indication. Patients requiring APAP may have a higher baseline symptom burden, such as pain from bone metastases or cancer-related fever, which itself reflects more aggressive disease biology and poorer prognosis. While most included studies employed multivariate analyses adjusting for key confounders—such as performance status, tumor burden, and line of therapy—the possibility of residual confounding from unmeasured factors (e.g., systemic inflammation or concurrent infection) cannot be fully excluded. Nevertheless, the consistency of the detrimental association across multiple independent cohorts, even after statistical adjustment, supports the likelihood of an independent effect of APAP. Future prospective studies with detailed documentation of indication-for-use and concomitant medications are warranted to further disentangle this relationship.

The dose–response relationship between APAP exposure and ICI outcomes has begun to emerge, lending further support to a potential causal link. Although high doses (e.g., 4 g/day) have been shown to induce Treg expansion in healthy volunteers (11), the immunologic effects of typical oncologic doses (1–2 g/day) require further clarification. Clinically, studies stratifying patients by plasma APAP concentration have demonstrated that higher levels at ICI initiation are associated with significantly worse survival outcomes (24, 27). Similarly, in real-world cohorts, high cumulative APAP exposure (e.g., ≥60 doses of 1000 mg) was identified as an independent predictor of shorter PFS and OS in NSCLC patients receiving ICIs, whereas low or short-term exposure showed no significant impact (27). Although the precise threshold for clinically relevant risk remains undefined, these findings suggest that both the magnitude and timing of APAP exposure—particularly sustained, high-dose use during early immune activation—may be key determinants of immunotherapy efficacy.

Notably, studies stratifying patients by APAP plasma concentration support a potential dose-response gradient, with higher levels at ICI initiation linked to significantly worse survival (24, 27). This implies that not only the presence but also the timing, dose, and systemic availability of APAP may modulate immunotherapy efficacy. In real-world settings, high cumulative APAP exposure (≥60 doses of 1000 mg) independently predicts shorter PFS and OS in NSCLC patients on ICIs, whereas low exposure (<24 hours or <60 doses) shows no significant impact, offering preliminary guidance for clinical practice (27).

The pooled hazard ratios of 1.29 for OS and 1.27 for PFS translate to an approximately 30% increase in the risk of death or disease progression among APAP users. This effect size is not only statistically significant but also clinically meaningful. In the field of immuno-oncology, a 30% increase in mortality risk represents a substantial effect, comparable to the detrimental impact observed with baseline corticosteroid or antibiotic use in patients receiving ICIs. This magnitude of risk underscores the need for heightened awareness and strategic management of concomitant APAP exposure. These findings also emphasize that even a widely perceived “safe” medication may compromise immunotherapy efficacy, highlighting the importance of incorporating medication review and pharmacovigilance into routine immunotherapy management to identify and mitigate modifiable risk factors.

Given these complexities, a risk-adapted approach to symptom management is warranted. For mild symptoms, non-pharmacologic measures such as physical therapy or cooling should be prioritized. When analgesia is necessary, short-term APAP use (<72 hours) may be acceptable in low-risk settings (e.g., during ICI maintenance or among strong responders), whereas NSAIDs with gastroprotection or low-dose opioids may be preferable in high-risk contexts (e.g., during early ICI cycles or in patients with low PD-L1 expression). Importantly, symptom control should not be compromised; analogous to WHO recommendations discouraging prophylactic APAP before vaccination to preserve immune responses, similar principles of timing and selective avoidance may apply in immunotherapy (28). NSAIDs, in particular, have shown improved OS compared to APAP in NSCLC patients receiving ICIs, though their use requires caution in those with renal or gastrointestinal comorbidities (16).

Several limitations of the primary studies merit consideration. First, retrospective designs risk residual confounding by indication, as patients requiring APAP may have higher symptom burdens reflective of aggressive disease. Second, variability in APAP exposure metrics—from plasma quantification to prescription records—precludes definitive dose-response conclusions. Third, insufficient data on concomitant medications (e.g., corticosteroids or antibiotics) limits assessment of drug-drug interactions. Finally, the predominance of NSCLC cohorts raises questions about generalizability to other malignancies.

Future research should address these gaps. Prospective studies quantifying APAP dose, timing, and indication relative to ICI cycles could identify vulnerability thresholds (e.g., >3 g/day within 48 hours of infusion). Biomarker-integrated studies measuring Treg or IL-10 dynamics in response to APAP may identify high-risk patients. Pragmatic trials comparing APAP-restrictive and permissive strategies—stratified by cancer type and co-medications—are ethically feasible using endpoints like pain control and ICI response. Regulatory updates, such as requiring drug labels to highlight APAP-ICI interactions, and documenting APAP exposure in electronic medical records, could facilitate real-world risk assessments and guide clinical decision-making.

## Conclusion

Current evidence indicates that APAP use is associated with poorer outcomes in ICI-treated cancer patients, likely via suppression of antitumor immunity. Clinicians should minimize unnecessary or prolonged APAP use during critical periods of immune activation, prioritizing alternative symptom management strategies when feasible. Further research is needed to establish causal relationships and define safe thresholds to guide evidence-based stewardship.

## Data Availability

The original contributions presented in the study are included in the article/**Supplementary Material**. Further inquiries can be directed to the corresponding author.
