# Supplementary material for: Acetaminophen use and prognosis in cancer patients treated with immune checkpoint inhibitors: evidence from a meta-analysis
Source: Front Immunol. 2025 Nov 21;16:1682686. doi: 10.3389/fimmu.2025.1682686 (PMC12678302; doi:10.3389/fimmu.2025.1682686)
Supplement: Supplementary file 1 [file Supplementaryfile1.docx]

**Supplementary Material**

**Acetaminophen use and prognosis in cancer patients treated with immune checkpoint inhibitors: Evidence from a meta-analysis**

**Supplementary Table 1.** Details of search strategy.

**Supplementary Table 2.** Quality assessment of included studies through the Newcastle-Ottawa Scale.

**Table S1** Details of search strategy.

| **Database** | **Search strategy** |
| --- | --- |
| ***PubMed*** | ((("Acetaminophen"[Mesh]) OR (Paracetamol)) OR (N-acetyl-para-aminophenol)) AND (((((((((("Immune Checkpoint Inhibitors"[Mesh]) OR ("Immune Checkpoint Blockers")) OR (PD-1)) OR (PD-L1)) OR (CTLA-4)) OR (Pembrolizumab)) OR (Durvalumab)) OR (Nivolumab)) OR (Atezolizumab)) OR (Ipilimumab)) |
| ***Web of Science*** | (Acetaminophen OR Paracetamol OR N-acetyl-para-aminophenol) (Topic) and ("Immune Checkpoint Inhibitors" OR "Immune Checkpoint Blockers" OR PD-1 OR PD-L1 OR CTLA-4 OR Pembrolizumab OR Durvalumab OR Nivolumab OR Atezolizumab OR Ipilimumab) (Topic) |
| ***Embase*** | ('acetaminophen'/exp OR paracetamol OR 'n acetyl para aminophenol') AND ('immune checkpoint inhibitors'/exp OR 'immune checkpoint blockers' OR 'pd 1' OR 'pd l1' OR 'ctla 4' OR pembrolizumab OR durvalumab OR nivolumab OR atezolizumab OR ipilimumab) AND ('overall survival'/exp OR os OR 'progression-free survival' OR pfs) |

**Table S2.** Quality assessment of included studies through the Newcastle-Ottawa Scale (NOS).

| **Studies** | **Selection** | | | | **Compatibility** | **Assessment** | | | **Total stars** | **Score** |
| --- | --- | --- | --- | --- | --- | --- | --- | --- | --- | --- |
|  | **Representativeness of the exposed cohort** | **Selection of the non-exposed cohort** | **Ascertainment of exposure** | **Demonstration that outcome of interest was not present at the start of study** | **Comparability of cohorts on the basis of the design or analysis** | **Assessment of outcome** | **Was follow-up long enough for outcomes to occur** | **Adequacy of follow up of cohorts** |  |  |
| Bessede et al 2022 | ★ | ★ | ★ | ★ | ★★ | ★ | ★ | ★ | 9★ | 9 |
| Ye et al 2020 | ★ | ★ | ★ | ★ | ★★ | ★ | ★ | ★ | 9★ | 9 |
| Nelli et al 2021 | ★ | ★ | ★ | ★ | ★★ | ★ | ★ | ★ | 9★ | 9 |
| Yamada et al 2021 | ★ | ★ | ★ | ★ | ★ | ★ | ★ | ★ | 8★ | 8 |
| Gobbini et al 2021 | ★ | ★ | ★ | ★ | ★★ | ★ | ★ | ★ | 9★ | 9 |

If six to eight factors of two cohorts were comparable, two stars were assigned; if four to five factors of two cohorts were comparable, one star was assigned; otherwise, no star was assigned. Studies assigned with score of seven to nine were defined as high methodological quality, while of five or six were moderate quality and of four or less were low quality. The score is equal to the total number of stars.
